# Who Pays the Heat Debt? Ethics, Labour, and Governance in a Warming World

**DOI:** 10.1111/bioe.70162

**Published:** 2026-07-15

**Authors:** Animesh Ghimire

**Affiliations:** ^1^ Faculty of Medicine, Nursing and Health Sciences Monash University Wellington Road Clayton Australia; ^2^ Sustainable Environment Foundation Nepal Kathmandu Nepal; ^3^ School of Public Health Chitwan Medical College Chitwan Nepal

**Keywords:** climate crisis, climate justice, global bioethics, heat debt, heat stress, labour rights, safety, worker health

## Abstract

Extreme heat is emerging as one of the most consequential occupational hazards of the 21st century. More than 2.4 billion workers, over 70% of the global labour force, are exposed to excessive heat, with more than 22 million heat‐related occupational injuries and nearly 19,000 deaths each year. By 2030, heat stress is projected to reduce global working hours by 2.2%, equivalent to 80 million full‐time jobs, with the heaviest losses concentrated in low‐ and lower‐middle‐income countries. This paper argues that these figures disclose not only a climate and labour crisis, but also a problem of health justice. To conceptualise this, the article develops the framework of ‘heat debt’ to articulate the physiological, economic, and temporal burdens disproportionately imposed on workers to sustain global production, supply chains, and essential services in a warming world. Synthesising evidence from agriculture, construction, garment manufacturing, informal urban labour, and healthcare, this paper demonstrates that this debt is systematically offloaded onto low‐wage, migrant, informal, and feminised workforces. The analysis proceeds in three parts. First, it argues that excessive occupational heat must be analysed through the lenses of distributive and reparative justice. Second, it demonstrates how current labour law, climate policy, and corporate supply chain governance institutionalise thermal inequality. Third, it interrogates health systems in their dual capacity: as primary responders to heat‐related morbidity, and as occupational environments that actively reproduce heat vulnerability among clinical staff. The article concludes by advancing a bioethical mandate of ‘cooling as care,’ positing that occupational thermal protection is an indispensable prerequisite for climate justice, decent work, and ethically defensible healthcare delivery.

## 1 Introduction: Heat, Work, and Invisible Costs

For billions of people, climate change is not experienced as graphs of rising global mean temperature, but as the fact that their working day has become intolerably hot. Recent International Labour Organisation (ILO) estimates suggest that around 2.4 billion workers—roughly 70% of the global workforce—are exposed to excessive heat at work, leading to about 22.85 million non‐fatal injuries, 18,970 deaths, and over 2 million disability‐adjusted life years (DALYs) annually [1, 2, 3]. A recent report from the World Health Organisation (WHO) and the World Meteorological Organisation (WMO) highlights the increasing frequency and intensity of extreme heat events. The report indicates that for each 1°C increase above a baseline of 20°C, worker productivity is projected to decline by 2%–3% [4, 5, 6]. Yet the significance of these estimates is not exhausted by epidemiological scale or macroeconomic loss. Occupational heat stress is also a test case for climate‐health ethics because it reveals how preventable bodily risks are allocated through organisation, labour regulation, migration status, and the structure of global production, rather than through environmental exposure alone [7, 8]. By 2030, heat stress is projected to reduce global working hours by 2.2%, equivalent to 80 million full‐time jobs and trillions of dollars in lost output, with the heaviest absolute losses in Southern Asia and West Africa [9].

Heat has become a leading cause of weather‐related mortality, with modelled estimates indicating around 489,000 heat‐related deaths each year between 2000 and 2019 [10, 11, 12]. This significant health burden is disproportionately concentrated in Asia and Africa, and these figures likely underestimate the actual impact. These global numbers, however, obscure a crucial ethical question: *whose bodies and whose work are paying the price for keeping economies, supply chains, and public services functioning in a hotter world?* This question is not only descriptive, as it asks how the benefits of continued production, consumption, and care are separated from the burdens of exposure, and therefore how duties of prevention, protection, and redress should be assigned. In this context, occupational heat is relevant to bioethics not only because it harms individuals, but also because it highlights a fundamental issue of health justice: the avoidable threats to health and well‐being are disproportionately experienced by those with the least power to protect themselves [13, 14].

I use the term *heat debt* to name this hidden ledger: the accumulation of physiological stress, illness, lost earnings, and early death borne by specific groups of workers so that others can live, consume, and profit under conditions of escalating heat. The concept has three interlocking dimensions. The first is *physiological debt*: the cumulative strain on the kidneys, cardiovascular system, nervous system, and mental health that results from repeatedly working near or beyond safe thermal limits. The second is *economic debt*: the lost wages, reduced work capacity, medical expenses, and reduced lifetime earnings that follow heat‐related illness and injury. The third is *temporal and intergenerational debt*: the transfer of today's inadequate mitigation, adaptation, and labour protections onto younger and future workers who will inherit more frequent and severe heat exposure [15]. Naming these dimensions at the outset clarifies that “heat debt” is not simply a metaphor for suffering; it is an analytical approach for connecting measurable harms to questions of responsibility.

The normative argument presented in this article posits that excessive occupational heat should primarily be understood through the lenses of distributive and reparative justice. It is distributive in nature because the burdens and benefits associated with heat exposure are unequally distributed among workers, sectors, regions, and generations. Furthermore, it is reparative since many of these burdens are foreseeable, systematic, and perpetuated by institutions such as governments, employers, supply chains, and health systems, which have the capacity to reduce exposure, compensate for losses, and redistribute the costs of adaptation [16, 17]. The objective here is not to assign blame, but to elucidate how empirical data regarding heat exposure creates ethical imperatives. This necessitates a comprehensive redesign of work processes, the allocation of resources for cooling solutions, the safeguarding of income during unavoidable work interruptions, and the active involvement of workers in developing safety standards. In what follows, I argue that if global bioethics is to take climate justice seriously, it must bring ‘heat debt’, and the governance that sustains it, into clear view.

## 2 Heat as an Ethical and Governance Problem

Heat is often characterised as an environmental condition that workers and employers must ‘adapt’ to. However, this framing is ethically misleading. The language of adaptation can obscure the reality that workplace heat exposure is influenced by factors such as wages, contracts, migration regulations, housing conditions, social protection, and the practical ability to slow down or cease work. Furthermore, heat serves as a medium of injustice, reinforcing and exacerbating existing inequalities related to income, gender, race, migration status, and geopolitical power [18, 19]. I use the term ‘medium of injustice’ in a structural context rather than as a pointed accusation: heat becomes unjust when institutions and market arrangements translate climatic conditions into predictable, preventable, and unequally distributed health risks. This perspective aligns with discussions of structural injustice, where responsibility is not reduced to identifying a single wrongdoer but is traced through social processes that allocate advantage, exposure, and vulnerability [20].

ILO projections show that by 2030, LMICs would be the worst affected, losing 4 and 1.5 per cent of their GDP in 2030, respectively, from heat‐induced reductions in labour productivity, while losses in Europe are close to zero [9]. Within the Asia–Pacific region, South Asia is expected to lose more than 5% of working hours in some countries, mainly in the agriculture and construction sectors, which are dominated by low‐wage, often informal workers [9, 21]. These projections are ethically significant because they show that occupational heat risk follows gradients of income, sector, and adaptive capacity, rather than temperature alone. Workers in middle‐ and low‐income tropical and subtropical regions face heightened risk precisely where formal controls, surveillance systems, and enforceable workplace protections are often least available [7, 22].

The unequal burden is also visible in mortality. Recent global assessments suggest that heat‐related deaths have increased by about 23% since the 1990s, reaching over half a million deaths annually, with the most significant risks in tropical regions [12, 23]. Recent studies attribute more than 500,000 heat‐related deaths over the last two decades to local warming from tropical deforestation, particularly in Southeast Asia, Africa, and the Amazon [24, 25]. Here, deforestation for commodities such as soy and beef externalises both climate risk and heat mortality onto rural communities with minimal political power. The relevant ethical consideration at this point transcends the notion that deforestation is harmful; it encompasses the intricate connections among land‐use decisions, commodity markets, and insufficient local protections, all of which link distant patterns of consumption to localised bodily risks. Consequently, heat transmission occurs through both the political economy and the atmosphere, highlighting the complex interplay between environmental degradation and socio‐economic structures.

Global trade makes this distributive structure more visible. A 2025 study indicates an 89% increase in labour exposure to extreme heat due to trade between 1995 and 2020 [26]. Lower‐middle‐income and low‐income economies account for approximately 72% of this exposure but receive only 7% of global labour compensation [26]. In essence, consumers and companies in high‐income countries benefit from goods produced by labour performed under hazardous heat conditions in other regions. This does not imply that all consumers or firms share equal responsibility. Instead, it highlights a structural pathway that delineates how the benefits of production are distributed and how the burdens of exposure are allocated across different areas. This understanding subsequently allows for responsibility to be articulated in terms of capacity, benefit, and institutional role, rather than relying solely on moral blame [20].

At the micro level, Parsons and colleagues conducted a mixed‐methods study across five occupational sectors in Cambodia, revealing that construction workers, garment workers, street vendors, agricultural labourers, and tourism workers often experience core body temperatures exceeding 38°C, above the threshold deemed safe. These workers also contend with low wages, insecure contracts, and limited access to healthcare. The researchers characterise this phenomenon as climate‐linked heat inequality [27]. These findings are significant as they illustrate the interplay between physiological exposure and social vulnerability. A worker's risk is influenced not only by ambient temperature but also by their ability to take breaks, access water and shade, decline unsafe tasks, seek medical treatment, and pursue compensation without losing their income.

From an ethical standpoint, these trends are not coincidental. They reflect deliberate governance choices related to labour law, social protection, urban planning, and corporate regulation. Many countries still lack enforceable occupational heat standards, do not acknowledge heat‐related illnesses as occupational diseases, and fail to provide income support when work must be halted due to heat [28, 29]. Systematic reviews on occupational health ethics, along with empirical research on ‘ethical climate,’ indicate that when organisations neglect to protect workers from foreseeable harms, they directly contribute to burnout, psychological distress, and moral injury [30, 31]. This section lays the empirical groundwork for the forthcoming normative analysis: heat debt emerges when predictable thermal risks coincide with a lack of voice, inadequate protection, and insufficient compensation. The deeper inquiry into what is owed to workers exposed to heat will be examined through the lenses of distributive and reparative justice.

## 3 Physiology and the Politics of Limits

The concept of heat debt extends beyond economic loss; it is also written into human physiology. Pioneering work by Sherwood and Huber [32] proposed that a wet‐bulb temperature (a measure of heat and humidity) of approximately 35°C represents a theoretical upper bound on human survivability. Beyond this point, even a person resting in the shade can no longer dissipate metabolic heat effectively through sweating and evaporation, and core temperature begins to rise. For many years, such conditions were treated as largely hypothetical. However, Raymond and colleagues [33] analysed observational data and found that short‐lived episodes approaching—and in some locations briefly exceeding—this threshold are already being recorded in parts of South Asia and the Persian Gulf, with more frequent exceedances projected as warming continues.

Crucially, a survivability threshold at rest is not an occupational safety threshold. Evidence from laboratory studies and modelling increasingly suggests that the critical limits for sustained activity occur at substantially lower wet‐bulb temperatures once real‐world factors are incorporated, including direct solar radiation, physical work intensity (metabolic heat production), clothing or protective equipment, and age [34, 35]. Vecellio and colleagues [36], for example, argue that practical limits for moderate work in humid environments may be closer to 30°C–31°C wet‐bulb. The reason is straightforward: while resting in shade adds little internal heat load, occupational tasks can rapidly tip the body into uncompensable heat stress because muscular work generates substantial metabolic heat and outdoor or industrial settings add radiant heat from the sun, hot surfaces, or machinery [37]. Under these conditions—such as brick workers exposed to intense radiant heat near kilns or farmworkers performing continuous exertion in direct sun—core temperature can rise quickly even when ambient conditions remain below the theoretical 35°C survivability threshold [19, 38].

Agricultural workers illustrate how physiological limits intersect with social vulnerability. In the United States and globally, research suggests that agricultural workers are roughly 35 times more likely to die from heat‐related illness than workers in other sectors [39, 40]. A joint Food and Agriculture Organisation and World Meteorological Organisation (FAO–WMO) assessment recently reported that 470 billion labour hours were lost globally in agriculture in 2021 due to extreme heat [41].

Qualitative reporting from India's brick kilns describes workers enduring surface temperatures far higher than the already extreme ambient air, experiencing dizziness and near collapse, yet continuing work to avoid wage loss [42]. In parts of Brazil, India, and the US Midwest, workers increasingly shift to night‐time schedules to escape daytime heat, but this disrupts sleep, family life, and safety, creating new forms of harm [43].

These examples underscore that the threshold at which work becomes ‘too hot’ is not purely a physiological fact. It is co‐produced by employment contracts, migration regimes, the presence or absence of unions, and social protection systems. Precarious and informal workers—disproportionately women, migrants, racialised groups, and those in the global South—have the least capacity to slow down, rest, or refuse unsafe work. The heat debt, therefore, accumulates fastest in bodies that are already devalued within broader social and economic hierarchies.

## 4 Heat, Care, and the Clinical Workforce

Health professionals usually encounter heat as a clinical problem: heat stroke among outdoor workers, acute kidney injury, or exacerbations of cardiovascular and respiratory disease during heatwaves [44]. Yet they are also workers within the same climate‐altered world, and increasingly, they deliver care in thermally unsafe conditions. The dual function of the clinical workforce positions it at the centre of ethical debates, as healthcare systems operate not only as entities responding to climate‐induced health issues but also as workplaces that contribute to environmental degradation. Recent scholarship in climate‐health ethics emphasises that bioethics should not only address bedside dilemmas but also consider the institutional, environmental, and infrastructural factors that influence the protection or deterioration of health [14, 45].

During the COVID‐19 pandemic and subsequent outbreaks, multiple studies found that wearing impermeable personal protective equipment (PPE) substantially increased heat strain in healthcare workers, worsening thermal discomfort, cognitive performance, and perceived safety [46, 47]. In one large retrospective study, the majority of PPE‐clad healthcare workers reported thirst, thermal discomfort, headaches, and reduced concentration during shifts in hot conditions [48, 49]. Experimental trials demonstrate that cooling vests and related technologies can significantly reduce perceived heat strain, sweat loss, and skin temperature among nurses and other staff in high‐heat environments [50]. Recent research on wearable sensor technology has demonstrated that exposure to heat and the use of PPE can lead to measurable physiological strain during simulated healthcare tasks. This evidence underscores that thermal risk in clinical environments transcends mere subjective discomfort; it represents a significant constraint impacting the safety and efficacy of work‐related activities [51].

Despite this, routine clinical settings—especially in low‐resource hospitals—often lack basic heat‐control systems. Wards have inadequate ventilation, no air‐conditioning or backup power, and poor thermal insulation, even as outdoor heatwaves intensify. The recent WHO report on workplace heat stress explicitly identifies health facilities as priority sites for prevention, arguing that protecting health workers from heat is essential for continuity of care [29]. The ethical importance of this issue lies not in the expectation that all health facilities can instantly achieve identical levels of thermal infrastructure. Instead, effective heat management has emerged as an essential component of the minimum institutional capacity required to provide safe and reliable care. A ward that becomes excessively hot, hindering sustained concentration, poses risks beyond mere discomfort; it jeopardises patient safety, staff wellbeing, and the conditions essential for exercising professional judgment.

The ethical concern is thus twofold. First, the professional obligations of beneficence and non‐maleficence cannot rest solely on individual clinicians, even as institutions overlook the conditions under which clinical care is provided. A climate‐conscious perspective on clinical ethics must consider whether health systems that mandate care in thermally unsafe environments are effectively shifting the burdens of beneficence onto workers' bodies. This extends recent arguments suggesting that health justice in the Anthropocene should incorporate the ecological and institutional factors that enable health, rather than treating environmental harms as separate from medical ethics [45, 52]. Second, given that the health workforce—particularly in nursing, community health work, and aged care—is predominantly female, the internal heat burden of health systems is also gendered: lower‐paid women often bear the highest thermal risks while having limited authority over infrastructure, staffing, uniforms, shift scheduling, or work‐rest arrangements [53].

Work on ethical climate and moral distress demonstrates that when workers perceive organisational neglect of safety and fairness, burnout, depression, anxiety, and turnover rise sharply [30, 31]. A systematic review examining the ethical climate within healthcare highlights that organisational norms significantly influence staff perceptions regarding what is deemed ethically acceptable, expressible, and actionable within clinical teams [54]. Normalising heat as ‘part of the job’ in hospitals and healthcare facilities, therefore, risks reproducing, within health systems, the very injustices that global health claims to address elsewhere. The bioethical implication is clear: health systems have the potential to perpetuate the very heat burden they aim to alleviate. Consequently, providing heat protection for clinical workers should not be viewed as a secondary employment benefit, but rather as a fundamental aspect of ethical care. The system's obligation to its patients cannot be justifiably upheld by undermining the health, agency, and moral integrity of those delivering care.

## 5 Governance and Accountability: Who Owes What to Whom?

If the heat debt is socially produced, it can be governed differently. The core governance challenge lies not just in the feasibility of cooling workplace environments but in determining the distribution of accountability among stakeholders involved in the creation, endorsement, or mitigation of hazardous heat exposure. Recent developments show both progress and the scale of the task. The new WHO–WMO joint guidance—*Climate change and workplace heat stress*—provides the first comprehensive global recommendations in over 50 years, calling for engineering controls (shading, ventilation, cool roofs), work‐rest cycles, hydration plans, early warning systems, and consideration of legal maximum working temperatures [5, 29]. In parallel, the ILO has launched a global campaign on heat stress, emphasising that prevention is both a labour right and an economic imperative [28]. These interventions matter because they shift heat from the realm of individual endurance to the domain of institutional design: exposure can be anticipated, measured, reduced, and compensated for.

However, gaps remain stark. Only a minority of countries have enforceable occupational heat standards; informal workers—street vendors, domestic workers, many farmworkers—are generally outside formal occupational safety and health (OSH) regimes [3]. Climate finance, national adaptation plans, and just transition frameworks rarely earmark resources for workplace cooling infrastructure or social protection that would allow workers to pause during extreme heat without incurring income loss. These omissions highlight the practical constraints of adaptation. A worker unable to take time off cannot classify heat illness as an occupational disease and, consequently, lacks access to compensation. While they are formally instructed to ‘adapt,’ the necessary conditions for safe adaptation are materially denied to them [44]. Climate justice scholarship proves valuable in this context, as it distinguishes between the various burdens of climate response, including prevention, adaptation, compensation, and institutional reform and examines which actors are best positioned or most obligated to bear each of these responsibilities [55].

Corporate governance, particularly in global supply chains, shows a similar blind spot. Investigations in the fashion sector report that garment workers in countries such as India and Bangladesh work in poorly ventilated factories where indoor temperatures during heatwaves approach 50°C, with symptoms of heat stress, kidney problems, and chronic fatigue [56, 57]. In 2023 alone, extreme heat is estimated to have cost 512 billion potential working hours, nearly 50% more than the 1990s average, yet very few brands have detailed policies or financing for heat adaptation in their supplier codes of conduct [58]. The accountability issue is intensified by the nature of supply chains: while purchasing firms may not have direct control over factory temperatures, their pricing strategies, lead times, audit practices, and contractual obligations can significantly influence whether suppliers perceive heat protection as feasible, unaffordable, or neglected. Consequently, heat exposure should be viewed as a value‐chain governance challenge rather than a local workplace hazard. Increasingly, scholarly discourse on due diligence acknowledges that adverse human rights and environmental impacts are often interwoven across production networks, necessitating that firms identify, prevent, mitigate, and account for risks that extend beyond the boundaries of their direct ownership [59, 60].

If corporations and consumers benefit from goods produced under dangerous heat conditions, I argue that they incur what might be called *heat responsibility*. This responsibility should be understood as differentiated rather than uniform. It does not imply that every actor bears the same duty, or that responsibility can be reduced to individual blame. Rather, responsibility should track at least four ethically relevant relations: contribution to heat risk, benefit from heat‐exposed labour, capacity to prevent or remedy harm, and institutional authority over the conditions of work. This approach aligns with accounts of structural responsibility, which locate duties not only in direct causation but also in participation and in social systems that predictably produce unjust outcomes [20]. On this account, the following commitments serve as essential benchmarks to evaluate whether governance frameworks meaningfully address heat debt:

1. Just adaptation financing: climate and development finance should explicitly allocate funds for worker‐centred cooling measures—shading, reflective roofing, ventilation, access to safe drinking water, rest facilities and cooling centres—along with social protection to buffer income loss when extreme heat disrupts work activities [26, 28]. This obligation falls most strongly on stakeholders with substantial adaptive capacity and those whose revenues depend on uninterrupted heat‐exposed labour.
2. Heat due diligence: firms should integrate metrics of heat exposure and rest‐break compliance into supply‐chain audits and environmental, social, and governance (ESG) reporting, treating extreme heat as a human‐rights risk alongside child labour and wage theft [26]. Such due diligence should extend beyond first‐tier suppliers to subcontracted, informal, and home‐based work where thermal risk is often least visible.
3. Legal accountability: states should adopt binding heat standards in labour law and recognise heat‐related illnesses (including chronic kidney disease linked to heat stress) as compensable occupational diseases, with evidentiary presumptions in favour of exposed workers [1, 61]. Legal recognition matters because it converts heat illness from an individualised misfortune into a publicly acknowledged occupational harm.

In the realm of bioethics, this governance analysis has significant institutional implications. Health systems, professional organisations, and academic institutions are not simply external observers of labour and environmental governance; they are also employers, purchasers, research sponsors, standard‐setters, and influential public advocates. Sustainable healthcare scholarship has already posited that health systems must consider the environmental and social conditions under which care is produced. The concept of heat debt extends this argument by emphasising the thermal safety of the workers who sustain care and its supply chains. As a bioethicist, I believe that institutions, journals, and funders have a critical role to play. They should advocate that climate adaptation, green transitions, and sustainable supply chains cannot be deemed ethical unless they address the thermal safety and compensation of the workers upon whom these systems rely.

## 6 Ethical Agenda: Cooling as Care and Recognising the Heat Debt

As previously mentioned, heat debt encompasses three dimensions, and its ethical implications become more evident when examined through the frameworks of distributive and reparative justice rather than as an ancillary list of policy issues. Increasingly, climate adaptation scholarship emphasises that justice involves not only distribution but also recognition, participation, and restoration. This perspective is particularly relevant here, as occupational heat not only creates risk but also distributes it through institutional frameworks, resulting in inequitable physiological, economic, and temporal effects [62, 63, 64].

### 6.1 As Noted Above, Heat Debt Has Three Interlocking Dimensions

- Physiological debt: the cumulative strain on kidneys, cardiovascular and nervous systems, and mental health, as well as the heightened risk of injury and premature death, borne by people who work year after year in temperatures approaching or exceeding their physiological limits [24, 34, 35].
- Economic debt: lost working days, reduced lifetime earnings, medical costs, and intergenerational poverty resulting from heat‐related illness, injury, and premature death—burdens that fall heaviest on already marginalised workers [9, 44].
- Temporal and intergenerational debt: today's emissions and failures of regulation and adaptation are effectively transferring decades of future heat exposure to younger generations. Estimates indicate that children born in 2020 will face a two‐ to sevenfold increase in extreme events, particularly heat waves, compared to individuals born in 1960, assuming current climate policy pledges remain unchanged [15].

These dimensions demonstrate why a framework of distributive justice is necessary, yet insufficient. Distributive justice interrogates how the benefits of production, consumption, and care are ultimately secured. Specifically, it asks whether these dividends rely on concentrating bodily harm and premature mortality among workers lacking the structural power to demand safety. However, where such harms are foreseeable, patterned, and historically sedimented through labour law, informalisation, global trade dynamics, and inadequate adaptation finance, reparative justice becomes equally imperative. In this context, reparative justice transcends retrospective compensation. It necessitates the institutional remediation of the systemic preconditions that normalise hazardous heat exposure as an inherent cost of livelihood generation, alongside the redistribution of resources essential for safe adaptation [65, 66, 67].

On this account, non‐maleficence and precaution in occupational design are not peripheral to justice, but rather its primary operational mandate. The acquisition of basic subsistence must not be predicated on enduring severe thermal risk; furthermore, under conditions of uncertainty, regulatory frameworks must prioritise pre‐emptive protection over retrospective compensation. Given that thermal vulnerability fluctuates according to work intensity, hydration, physiological status (such as pregnancy, age, and chronic illness), acclimatisation, and access to rest, protective thresholds must be conservative and explicitly calibrated to accommodate different susceptibility [34, 35, 68, 69].

The integration of worker voice, participation, and recognition forms the second operational requirement. Top‐down heat standards routinely fail to identify the true systemic loci of risk production, overlooking critical factors such as piece‐rate wages, retaliatory management practices, immigration precarity, substandard housing, and the economic unviability of unpaid breaks. Participatory heat interventions have demonstrated clear efficacy in improving risk awareness, aligning with adaptation‐justice literature that positions procedural and recognitional justice at the core of effective climate governance. Therefore, populations with heightened physiological or social vulnerability, including pregnant individuals, migrant workers, and those with pre‐existing illnesses, must not be treated as policy addenda, but rather as the critical benchmark for assessing whether occupational protections are fundamentally equitable [62, 70, 71].

Within health systems and global bioethics, this analysis points to a critical imperative: ‘cooling as care.’ This phrase operates not merely as a metaphor, but as a literal and institutional mandate. Care is not confined to interpersonal bedside virtues; it is structurally enacted through the infrastructures that facilitate safe attention, sound judgment, and effective treatment. Contemporary bioethical discourse increasingly argues that climate ethics must move beyond the micro‐level duties of individual clinicians to address the macro‐level obligations of healthcare systems, including mitigating environmental and occupational hazards. A care‐ethics perspective reinforces this shift by emphasising human interdependence, physiological vulnerability, and the resources necessary for equitable healthcare delivery [72, 73, 74, 75].

Viewed through this lens, remediating the heat debt necessitates a coordinated matrix of institutional obligations. States must codify binding thermal standards, wage protection, and accessible compensation pathways for heat‐related illness within labour law. Employers and lead firms must finance the material infrastructure of cooling—such as shade, ventilation, hydration, rest, resilient power grids, and physiologically safe shift designs—rather than externalising these costs onto a precarious workforce. Health systems must conceptualise thermal safety as a fundamental component of ethical care infrastructure, investing in passive cooling, reliable energy, adequate staffing, and PPE protocols that mitigate heat strain. Finally, clinicians and public health professionals must champion occupational thermal protection as a core tenet of health justice and decent work, rather than a peripheral technicality of climate adaptation.

## 7 Conclusion: Paying Down the Heat Debt

Extreme heat is often described as an invisible threat, yet its impacts are unevenly distributed across society. Those who harvest crops, sew garments, work in construction, and provide care are not mere collateral damage of a warming climate; they form the essential workforce upon which modern economies increasingly rely for their resilience to heat. What this paper conceptualises as ‘heat debt’ articulates this dependency precisely: it encompasses the physiological damage, economic loss, and temporal displacement of risk that enable production, circulation, and care to continue amid rising temperatures. So long as this debt remains absent from adaptation frameworks, labour law, trade governance, and institutional ethics, climate justice will remain rhetorically compelling but materially incomplete.

The central ethical question is not simply how to cool individual workplaces, but how to rectify an economic order that normalises preventable heat exposure as a condition of employment. Evaluated through distributive and reparative justice, occupational heat reveals a patterned ethical deficit: the socioeconomic benefits of work performed in dangerous heat are widely shared, whereas the health impacts are concentrated among those with the least agency to refuse unsafe conditions. Informal workers, migrants, low‐wage labourers, and those in undervalued care roles do not just face higher temperatures; they are structurally denied the protections, compensation, and procedural voice required to make heat exposure governable.

Consequently, the ‘heat debt’ framework brings physiology, labour rights, climate governance, and bioethics into a cohesive analytical frame, proving that these domains cannot be ethically siloed. Once heat debt is rendered visible, responsibility ceases to be discretionary. States must embed binding heat standards, income protection, and compensable recognition of thermal illness into law. Corporate stakeholders must absorb thermal safety as a fundamental cost of production rather than displacing it down the supply chain. Furthermore, health systems must adopt ‘cooling as care’ as an institutional mandate, acknowledging that ethical patient care cannot rely upon the compromised health and agency of its workforce.

For bioethics, extreme heat represents a fundamental test of the field's capacity to confront the infrastructural conditions under which health is negotiated. A bioethical framework suited to the climate crisis must look beyond acute emergencies to address the routine, normalised harms through which unjust systems operate. In a hotter century, the clearest indicator of justice will be whether societies continue to extract prosperity from the thermal vulnerabilities of marginalised populations or finally commit to paying down the debt.

## Author Contributions

Conceptualization: Animesh Ghimire. Formal analysis: Animesh Ghimire. Investigation: Animesh Ghimire. Writing – original draft preparation and writing – review and editing: Animesh Ghimire. The author approved the final version to be published.

## Funding

The author has nothing to report.

## Ethics Statement

The author has nothing to report.

## Conflicts of Interest

The author declares no conflicts of interest.

## Data Availability

The author has nothing to report.
